# SNHG3 Functions as miRNA Sponge to Promote Breast Cancer Cells Growth Through the Metabolic Reprogramming

**DOI:** 10.1007/s12010-020-03244-7

**Published:** 2020-01-20

**Authors:** Yan Li, Zhenhui Zhao, Wei Liu, Xun Li

**Affiliations:** Breast Internal Medicine Department, The 3rd Affiliated Teaching Hospital of XinJiang Medical University (Affiliated Cancer Hospital), Urumqi, 830011 China

**Keywords:** SNHG3, Cancer-associated, Fibroblasts, miR-330, Metabolism reprogram, Breast cancer

## Abstract

Cancer-associated fibroblasts (CAFs) are important ingredient in tumor microenvironment. The dynamic interplay between CAFs and cancer cells plays essential roles during tumor development and progression. However, the mechanisms of intercellular communication between CAFs and cancer cells remain largely unknown. We characterized exosomes secreted from breast cancer patient-derived CAFs by transmission electron microscopy. The expression of SNHG3, miR-330-5p, and PKM (Pyruvate Kinase M1/M2) was examined by real-time QPCR and immunoblot. The function of SNHG3 on the growth and metabolism of tumor cells was used by CCK8 and mitochondrial oxygen consumption assays. The binding between SNHG3, miR-330-5p, and PKM was examined by dual luciferase reporter assays. Orthotopical xenograft of breast tumor experiments was performed to determine the function of SNHG3 in vivo. We demonstrated that exosomes secreted from CAFs reprogram the metabolic pathways after tumor cells uptake the exosomes. CAF-secreted exosomal lncRNA SNHG3 served as a molecular sponge for miR-330-5p in breast cancer cells. Moreover, PKM could be targeted by miR-330-5p and was controlled by SNHG3 in breast cancer cells. Mechanistically, SNHG3 knockdown in CAF-secreted exosomes suppressed glycolysis metabolism and cell proliferation by the increase of miR-330-5p and decrease of PKM expression in tumor cells. SNHG3 functions as a miR-330-5p sponge to positively regulate PKM expression, inhibit mitochondrial oxidative phosphorylation, increase glycolysis carboxylation, and enhance breast tumor cell proliferation. Overall, SNHG3 could play a major role in the development and progression of breast cancer and support the therapeutic potential of targeting communication between cancer cells and tumor microenvironment.

## Introduction

Tumor microenvironment (TME) consists of various cell types including cancer-associated fibroblasts (CAFs), tumor cells, and inflammatory cells [1, 2]. In cancer stroma, normal fibroblasts have been transformed into CAFs, which are one of the major components of the TME in many solid tumors. More and more research revealed that CAFs in the TME could not only be recruited but also be activated by paracrine factors released from tumor cells, which enable a molecular communication between CAFs and cancer cells, releasing large numbers of cytokines and regulating tumor growth and metastasis [1, 2]. Although CAFs have been associated with tumor cell proliferation, metabolism, angiogenesis, and metastasis [1, 2], little is known about their specific roles of intercellular communications with cancer cells and the underlying mechanisms in the development and progression of cancers. Therefore, the investigation of underlying mechanisms between tumor and stromal cells like CAFs in the TME is essential in generating new therapeutic methods that can prevent cancer development and progression [3–5].

Tumor cells and CAFs form a dynamic interaction network in the tumor microenvironment [2]. Extracellular vesicles secreted from cancer cells (such as exosomes) were demonstrated as an important intercellular communication. Exosomes could deliver various biomolecules among different cells by moving in the intracellular area [6]. Cancer-secreted exosomes are associated with cancer progression, angiogenesis and immune exhaustion. Exosome-encapsulated noncoding RNA (miRNA or lncRNA) in cancer-secreted exosomes could regulate gene expression and signaling pathway in a post-transcriptional manner in niche cells [6, 7]. Previous studies focused heavily on tumor cell secreted exosomes; however, CAF-derived exosomes and their functions on tumor cells remain largely elusive. Although increasing evidence suggests that CAFs can secrete exosomal non-coding RNA (ncRNA) to promote the growth of tumor cells [6, 7], the contribution of CAF-secreted exosomal ncRNA in the development and progression of cancer has not been elucidated. Consequently, there is a growing therapeutic interest to investigate the communication mechanisms between CAFs and tumor cells in TME and to identify novel targets for cancer treatment.

Long non-coding RNAs (lncRNAs), a group of RNA, are 200 nucleotides lengths without a complete open reading frame (ORF) [8, 9]. LncRNAs could be transcribed by RNA polymerase, spliced, and modified in the nuclei which was similar to mRNAs transcription [8, 9]. Current studies have revealed that the malfunction of lncRNA expression resulted in the progression of different tumors because the lncRNAs could function as oncogenes or tumor suppressor genes [8, 9]. As lncRNAs get involved in many biological functions such as carcinogenesis, researchers paid increasing attentions on the study of lncRNAs [8, 9]. Small nucleolar RNA host gene 3 (SNHG3) was discovered as a new lncRNA, which located on 1q35.3 [10]. Accumulating evidence demonstrates that the expression of SNHG3 was increased in variety types of tumor tissues such as breast cancer, hepatocellular carcinoma, and colorectal cancer, resulting in increased proliferation and metastasis of tumor cells and poor survival of tumor-bearing patients [10–13]. LncRNA was able to be released to the extracellular spaces by exosomes or in protein complexes or lipid carriers [14]. Previous studies showed that lncRNA could be secreted into extracellular space and regulate the function of neighboring or distant cells in a paracrine manner [15]. Therefore, extracellular lncRNA is considered as a novel type of messengers and effectors in intercellular cross talk. However, the regulatory roles and the detailed mechanism of CAF-secreted exosomal SNHG3 in breast cancer remain poorly understood. We hypothesized that lncRNAs contained within CAF-secreted exosomes can drive the modulation of metabolic activities in breast cancer cells and that exosomal lncRNAs serve as important factors for re-programming the tumor microenvironment.

In our study, we explored both the expression patterns and biological functions of breast cancer derived CAF-secreted exosomal SNHG3 on breast tumor cells, as well as the molecular mechanism of SNHG3 during the development of breast cancer. The results demonstrated a new metabolic regulatory function of CAF-secreted exosomal lncRNA in breast cancers. More importantly, we provided a novel regulation pathway between tumor cells and tumor microenvironment, which may offer novel targets for cancer therapy.

## Materials and Methods

### Cell Culture and Transfection

MCF-7 and MD-MBA-453 breast cancer cells were purchased from the American Type Culture Collection (ATCC; Maryland, MD, USA). MCF-7 and MD-MBA-453 cells were respectively cultured in ATCC-formulated Eagle’s minimum essential medium and ATCC-formulated Leibovitz’s L-15 medium plus 10% fetal bovine serum (FBS; Gibco, Thermo) and antibiotics (100 U/ml penicillin and 100 μg/ml streptomycin sulfate) (HyClone, USA) at 37 °C in a humidified incubator with 5% CO_2_. Cells at 75% confluence were harvested for each experiment.

Breast cancer patient–derived fibroblast cells were maintained in Iscove’s modified Dulbecco’s medium adding 15% FBS. CAFs were seeded in 15 cm dish and when the CAFs reached 75% confluent. CAF-secreted exosomes were isolated from the culture medium of CAFs after 48 h, and then exosomes were added into the medium for continuous culture of breast cancer cells.

siRNAs against SNHG3, siRNA control (si-con), SNHG3 (SNHG3), Pyruvate kinase isozymes M1/M2 (PKM), empty vector (Vector), miR-330-5p mimic (miR-330), miRNA scrambled control (miR-con), miR-330-5p AntagomiR (anti-miR-330), and AntagomiR control (anti-miR-con) were generated from Genscript (Nanjing, China). Transfection was performed using Lipofectamine 2000 (Invitrogen) with various oligonucleotides or plasmids. Cells were harvested 48 h after transfection for further experiments.

### Exosome Extraction

Cells were cultured with exosome-depleted serum, which was prepared by centrifugation of FBS at 100,000×*g* at 4 °C. Then, we collected the culture medium and centrifuged after 72 h incubation. Floating cells were removed from the medium following a centrifugation at 400×*g* for 5 min at 4 °C. Next, cell debris was further removed from the supernatants by centrifugation at 3000×*g* for 20 min at 4 °C. After flirtation of the supernatants by passing through a 0.22-μm filter, exosomes in the supernatants were ultracentrifuged and collected at 110,000×*g* for 4 h at 4 °Cusing ultracentrifuge (Beckman). After wash of PBS, exosomes were stored at *t* − 80 °C for further experiments.

### Exosome Size Distribution Measurement

Exosomes were assessed for size distribution using a Nanobrook Omni (Brookhaven). Exosomal sizes and distribution were measured after they were resuspended and diluted in PBS by adding 2 ml of exosomes PBS into the Nanobrook Omni system.

### TEM

The morphology of exosome samples was assessed by TEM. First, we prepared and diluted exosomes in PBS and place exosome-containing liquid on the copper grids. Then, the copper grids were dried and the excessive liquid was removed. Next, the samples were stained by 2% phosphotungstic acid (PTA) for 5 min at r.t. and fixed with 2% glutaraldehyde for 5 min. After PBS washing for three times, exosomes were imaged by transmission electron microscope (JEM-1230, Japan).

### Exosome Labeling and Uptake by MCF-7 and MD-MBA-453 Cells

Exosomes secreted from the breast-derived CAFs were isolated as described above. After washing by PBS, the exosomes were stained by PKH67 agent (Sigma) according to the manufacturer’s instructions after an ultracentrifugation at 120,000×*g* for 4 h at 4 °C. Exosomes without PKH67 staining or no exosome adding were selected as the negative controls. To investigate the uptake of exosomes by cancer cells, MD-MBA-453 and MCF-7 cells were seeded in confocal imaging chamber. After a 24-h culture period, the chamber was washed by PBS for three times and cells were stained with different medium with either PKH67-labeled exosomes or blank control. After a further incubation for 48 h, each confocal chamber was washed by PBS for three times and cells were fixed by 4% PFA for 8 min. The DNA was stained using DAPI and washed by PBS for another two times. Finally, the uptake of exosomes by tumor cells were imaged by confocal microscope LSM880 (Carl Zeiss, Germany) and the images were further analyzed using Zen software (Carl Zeiss, Germany).

### Viability Assay

CCK8 assay was performed suing CCK8 detecting kit (Dojindo) for the assessment of cell viabilities according to the manufacturer’s instructions. Briefly, cells were plated in 96-well plate (Corning) in each condition. After an incubation with CCK8 assay solution for 2 h, the absorbance was recorded at the length of 450 nm.

### OCR and ECAR Measurements

Oxygen consumption rate (OCR) and extracellular acidification rate (ECAR) were determined by the XF metabolic analyzers (Sea-horse, Agilent). First, we plated cells in Seahorse 24-well microplates. After the density of cells reached 70% confluent, each culture medium with indicated conditions was added in each well. Then, the plate was replaced by 800 μL of assay media after 12 incubation at 37 °C with 5% CO_2_. The OCR was measured after another 1 h incubation at 37 °C without 5% CO_2_. The measurement of ECAR was similar the OCR assay. The normalization of each OCR or ECAR value was calculated by cellular protein mass.

### RNA Extraction and Real-Time QPCR

RNA was isolated from MD-MBA-453 cells by Trizol (Thermo), and cDNA was generated by reverse transcription kit (TaKaRa, China). 36B4 (human) was selected as an internal control. Real time QPCR was processed by the SYBR Green Mix (Yeasen, China). Data was acquired and analyzed by StepOnePlus Real-Time PCR System (Thermo).

### Western Blot

The cell protein was extracted using RIPA buffer (Thermo). Protein concentration was determined by BCA Protein Assay (Beyotime). Total protein was loaded, fractionated by SDS-PAGE, transferred to PVDF membrane, and probed with anti-β actin (Abcam), anti-PKM (Abcam), anti-PFKM (Cell Signaling Technology), anti-PDHB (Abcam), and anti-IDH2 (Cell Signaling Technology). Signal was detected using Chemiluminescence imaging system (Tanon, Shanghai, China).

### Generation of Stable Cell Lines by Lentivirus Transduction

shRNAs binding the sequence of SNHG3 (sh-SNHG3), scrambled control (sh-control), anti-miR-330-5p (anti-miR-330), and anti-miR-control (anti-miR-con) were cloned into pCDH vectors containing red fluorescent protein (mcherry) reporter. Briefly, these plasmids were transfected into HEK293T cells, along with helper plasmids as packaging vectors (GeneChem, Shanghai, China). MD-MBA-453 cells were transduced with a lentivirus containing anti-miR-330-5p using the lentivirus and 5 mg/ml polybrene Genechem (Shanghai, China). FACS analysis was performed 2 days after the transfection with the lentivirus for measuring mCherry signal to obtain cells with stably miR-330-5p knockdown. Similarly, breast cancer patient–derived CAFs were transduced with sh-SNHG3 expressing lentivirus to obtain stably SNHG3 knockdown cells.

### Luciferase Reporter Assay

Plasmid including the luciferase reporter genes was constructed by inserting SNHG3 fragments composed of the control and mutation of miR-330-5p binding sites into firefly luciferase backbone plasmids (pGL3, Promega). pGL3-PKM 3′-UTR was generated by cloning the 3′-UTR sequence of PKM into pGL3 plasmids. Equal numbers of MD-MBA-453 cells were transfected with 250 ng SNHG3-WT or SNHG3-Mutation, 25 ng of renilla reporter plasmid, miR-330-5p, anti-miR-330-5p, or mismatched controls, or cotransfected with PKM 3′-UTR and miR-con, miR-330-5p, miR-330-5p + plasmids, miR-330-5p + PKM, anti-miR-con, anti-miR-330-5p, anti-miR-330-5p + si-control, or anti-miR-330-5p + si-SNHG3. Cell lysates were collected and diluted using Dual-Luciferase Reporter Assay system (Promega).

### Generation of Breast Cancer Mouse Model

Female BALB/c nude mice weighing 20 g were acquired from XinJiang Medical University (XinJiang, China). To generate the breast cancer bearing mouse model, 5 × 10^6^ MD-MBA-453 alone or stably expressing anti-miR-330-5p cells (MD-MBA-453/anti-miR-330-5p or MD-MBA-453/control), or mixed with 1 × 10^6^ CAFs expressing shSNHG3 or control (CAF/shNHG3 or CAF/control), that were mixed at 1:1 with Matrigel (Thermo) were inoculated in the right, fourth mammary fat pads of the nu/nu mice. Upon reaching average tumor volumes of 50 mm^3^, the animals were randomly separated into indicated groups. Intratumoral pH was measured by a pH meter (Thermo). The animal experiment was approved by the Ethics Committee of Xinjiang Medical University (No: XJMU20190012).

### Measurements of Lactate and Acetate Levels

The metabolic profiling including the levels of lactate and acetate was examined by kits according to the manufacturers’ protocols, including acetate assay kit (Thermo) and the lactate assay kit (Thermo).

### Statistics

All results were displayed as mean ± s.e.m. from at least three independent experiments. The analyses were performed for significance by using Prism software (GraphPad 8). Unpaired Student’s *t* test and one-way analysis were used for the analysis of significant differences between two groups. *P < 0.05, **P < 0.01, and ***P < 0.001 were considered as significant.

## Results

### The Uptake of CAF-Secreted Exosomes by Breast Tumor Cells

**Fig. 1:**
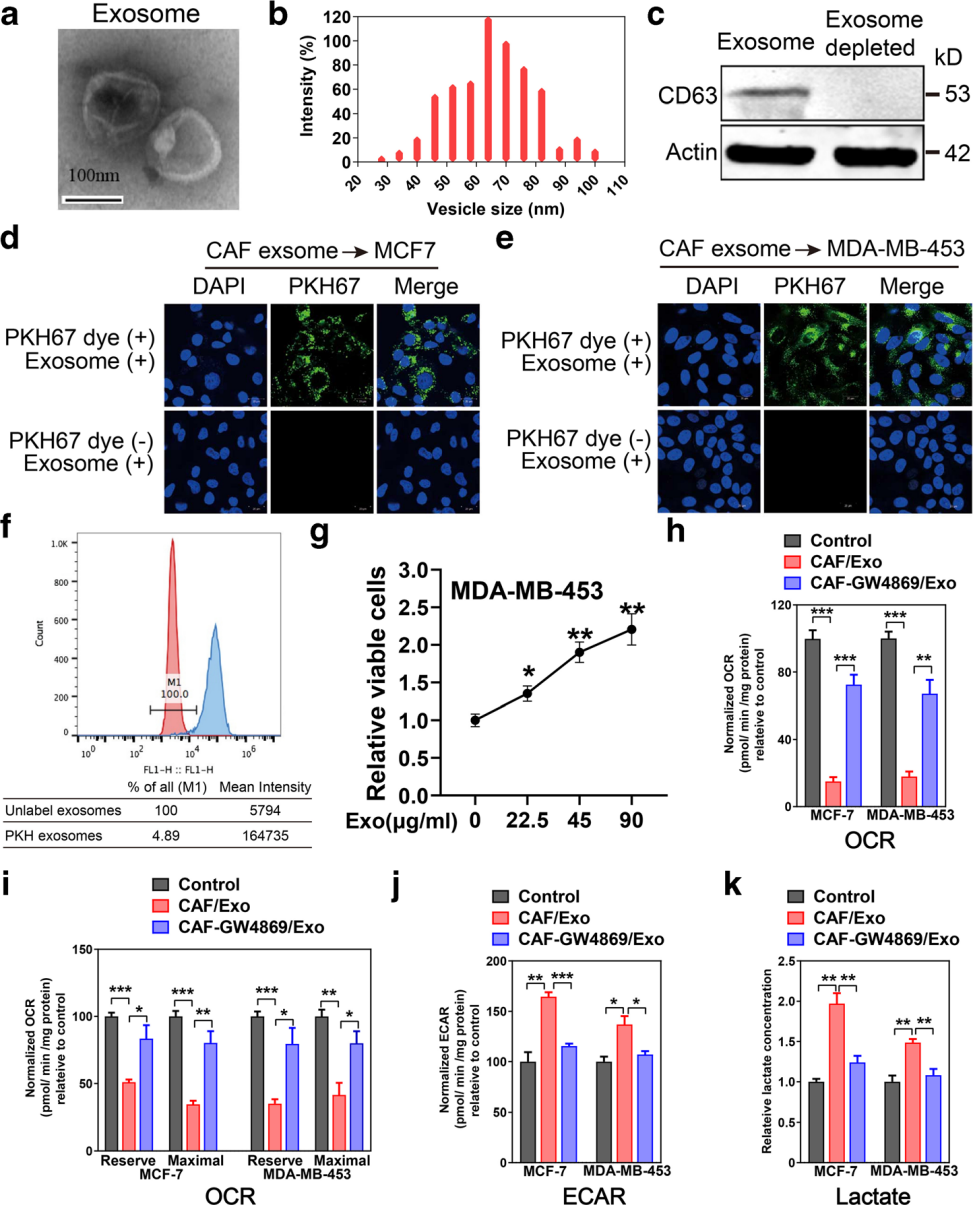
CAF-derived exosomes reprogrammed the metabolism of breast cancer cells. **a** Transmission electron microscopy (TEM) images of exosomes secreted from CAFs. **b** Hydrodynamic sizes of exosomes isolated from CAFs. **c** Western blot analysis of CD63 expression in CAF-secreted exosomes. **d**, **e** Uptake of CAF-secreted exosomes secreted from the two breast cancer cell lines into MCF7 and MD-MBA-453 cells. Exosomes secreted from the CAFs were stained with PKH67 dye (green signals). Cells were imaged by a confocal microscope. **f** Flow cytometry analysis revealed the uptake of CAF-secreted exosomes by MD-MBA-453 breast tumor cells. MD-MBA-453 cells were cultured with PKH67-labeled CAF-secreted exosomes for 4 h. **g** Viability of breast cancer cells after the treatment of CAF-secreted exosomes for 48 h (MD-MBA-453) (*n* = 9). **h** Basal OCR were measured for MCF-7 and MD-MBA-453 cells cultured with breast cancer secreted CAFs exosomes. OCR of both MCF-7 and MD-MBA-453 were downregulated by CAF- secreted exosomes. **i** Maximal and reserve OCR of MCF-7 and MD-MBA-453 were examined after the uptake of CAF exosomes (*n* = 12). **j** ECAR of both MCF-7 and MD-MBA-453 cells were determined after the uptake of CAF-secreted exosomes (*n* = 12). **k** Lactate concentration were assayed by GC-MS in MCF-7 and MD-MBA-453 cells incubated with or without CAF-secreted exosomes for 24 h. GW4869 was used to inhibit the exosome generation

To investigate that whether CAFs could secrete exosomes, and the uptake of CAF-secreted exosomes by cancer cells, the exosomes were extracted from culture medium released from CAFs obtained from breast cancer bearing patient. Transmission electron microscope was used to examine the morphology of exosomes secreted by CAFs and exosomes showed round-shaped structure with the typical cup-shaped structure (Fig. 1a).

Consistent with previous studies [7], the particle size of CAF-secreted exosomes ranged from 40 to 100 nm (Fig. 1b). Then, we confirmed the expression of exosome marker CD63 through western blot (Fig. 1c). To examine if CAF-secreted exosomes were taken up by breast cancer cells (MCF-7 and MD-MBA-453), we pre-stained exosomes with PKH green dye and subjected exosomes to MCF-7 or MD-MBA-453 cells. Green fluorescent signals were found in MCF-7 or MD-MBA-453 cells through the confocal laser scanning microscopy (Fig. 1d, e). The uptake of CAF-secreted exosomes by breast cancer cells was further confirmed by flow cytometry. The results conformed the uptake of CAF-secreted exosomes by breast tumor cells (Fig. 1f), indicating that exosomes derived from CAFs are taken up into breast cancer cells.

### Mitochondrial Function of Breast Tumor Cells Was Inhibited by CAF-Secreted Exosomes

Previous studies showed that CAFs could control tumor cell proliferation [1, 2]; we first verified the effect of CAF-secreted exosomes on tumor cell proliferation. Exosomes secreted from the medium of CAFs from a breast cancer patient were isolated and used to culture breast cancer cells in the presence of isolated exosomes. CAF-secreted exosomes increased the growth of MD-MBA-453 cells in an exosomes-concentration dependent manner (Fig. 1g). To examine whether CAF-secreted exosomes induce metabolic reprogramming in tumor cells, we cultured MD-MBA-453 cells with CAF-secreted exosomes and detected the oxygen consumption rate (OCR). The results showed that OCR of both MCF-7 and MD-MBA-453 cells were decreased in presence of breast CAF-secreted exosomes. To verify whether this reduction of OCR in tumor cells indeed resulted from the internalization of exosomes, we blocked the generation of exosomes with GW4869 (exosome-release inhibitor) in CAFs. Notably, GW4869 could partially reverse this decrease of OCR in breast cancer cells, indicating that the CAF-secreted exosomes mediated the reduction of OCR in tumor cells (Fig. 1h). We founded that maximal and reserve mitochondrial function of tumor cells were markedly decreased treated with exosomes, suggesting that mitochondrial respiratory capacity was suppressed by CAF-secreted exosomes (Fig. 1i). Moreover, ECAR (extracellular acidification rate) increased significantly in breast tumor cells when co-cultured with CAF-secreted exosomes (Fig. 1j). Lactate levels also increased significantly in the breast tumor cells in presence of exosomes (Fig. 1k). Taken together, our results showed that CAF-secreted exosomes decreased mitochondrial function and reprogrammed metabolic pathways of breast tumor cells.

### CAF-Secreted Exosomal SNHG3 Promoted Proliferation and Downregulated Mitochondrial Role in Breast Tumor Cells

**Fig. 2:**
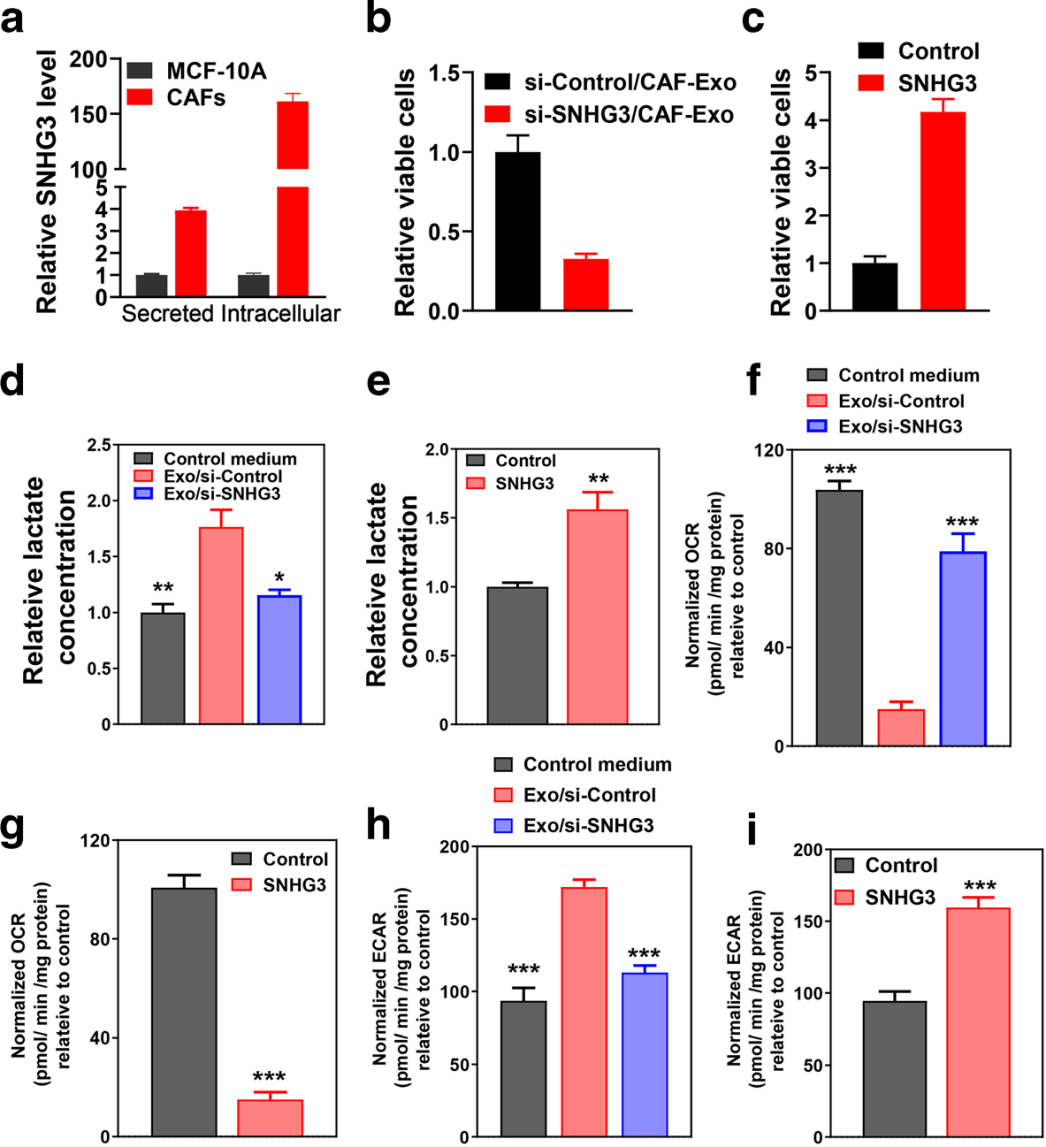
CAF-derived exosomal SNHG3 reprogrammed the metabolism of breast cancer cells. **a** SNHG3 is secreted highly by breast tumor derived CAFs. RNA was isolated from exosomes in the culture media supernatant after ultracentrifugation at 110,000×*g* and analyzed for SNHG3 by real-time quantitative PCR. Data were normalized to amounts of total proteins (secreted) or 36B4 (intracellular). The normal breast cell line MCF10A was selected as negative control (*n* = 6 extracts). **b** Cell viability was determined by the CCK8 assay at 72 h in MDA-MB-453 cells treated with CAF-derived exosomes transfected with si-SNHG3 or si-control. **c** The CCK8 assay was performed to detect cell viability at 72 h in MDA-MB-453 cells transfected with SNHG3 overexpression plasmid. **d** Relative lactate levels were assessed by GC-MS in MD-MBA-453 cells treated with CAF-derived exosomes transfected with si-SNHG3 or si-control for 24 h (*n* = 4). **e** Lactate concentrations were determined through GC-MS in MD-MBA-453 cells transfected with SNHG3 overexpression plasmid. **f**, **g** Basal OCR was measured for MD-MBA-453 cells treated with CAF-derived exosomes transfected with si-SNHG3 or si-control or MD-MBA-453 cells transfected with SNHG3 overexpression plasmid. **h**, **i** Basal OCR was measured for MD-MBA-453 cells treated with CAF-derived exosomes transfected with si-SNHG3 or si-control or MD-MBA-453 cells transfected with SNHG3 overexpression plasmid

Noncoding RNAs (e.g., lncRNAs) in the exosomes could function as a cross talk mechanism between stromal and tumor cells [16, 17]. As we have demonstrated the exosomes secreted from CAFs could promote proliferation and reprogram metabolism of breast tumor cells, we further examined whether the effect is dependent on CAF-secreted exosomal lncRNA SNHG3. We first examined both secreted and intracellular expression level of SNHG3 in CAFs. Breast cancer derived CAFs secreted significantly increased SNHG3 than that of normal breast cells MCF10A (Fig. 2a). Then loss-of-function and gain-of-function assays were carried out to investigate the biological function of SNHG3 during the growth of breast cancer cells. CCK8 assays were utilized to identify the alteration of cell proliferation in MDA-MB-453 cells treated by exosomes secreted from CAFs transfected with si-SNHG3 or with SNHG3 overexpression plasmid. When compared with the si-control group, MDA-MB-231 cells treated by exosomes secreted from CAFs transfected with si-SNHG3 exhibited a significant inhibition of proliferation by the CCK8 assays (Fig. 2b). Consistently with this, MDA-MB-453 cells directly transfected with SNHG3 overexpression plasmid markedly enhanced the proliferation compared with control group (Fig. 2c). Furthermore, the treatment of exosomes secreted from CAFs transfected with si-SNHG3 rescued the enhancement of lactate production (Fig. 2d), while overexpression of SNHG3 in MDA-MB-453 cells significantly increased lactate production (Fig. 2e). Then, we examined mitochondrial respiration in breast cancer cells with or without exosomes secreted from CAFs transfected with si-SNHG3. OCR of MDA-MB-453 cells decreased treated by CAF-secreted exosomes and could be rescue in the presence of exosomes secreted from CAFs transfected with si-SNHG3 (Fig. 2f). Consistent with the decreased OCR treated by CAF-secreted exosomes, overexpression of SNHG3 in MDA-MB-453 cells significantly suppress oxygen consumption rate (Fig. 2g). Furthermore, we examined the glycolysis level in breast tumor cells co-cultured with exosomes secreted from CAFs transfected with si-SNHG3. The increase in ECAR of MDA-MB-453 cells treated with CAF-secreted exosomes could be rescued by the knockdown of SNHG3 in CAFs (Fig. 2h). The increase of ECAR when MDA-MB-453 cells overexpressing SNHG3 (Fig. 2i) further confirmed that the alteration of glycolysis metabolism in breast cancer cells is dependent on CAF-secreted exosomal SNHG3. Collectively, these results indicated CAF-secreted exosomal SNHG3 could promote proliferation and downregulate mitochondrial function in breast cancer cells.

### CAF-Secreted Exosomal SNHG3 Regulates the miR-330 Expression in Breast Tumor Cells

**Fig. 3:**
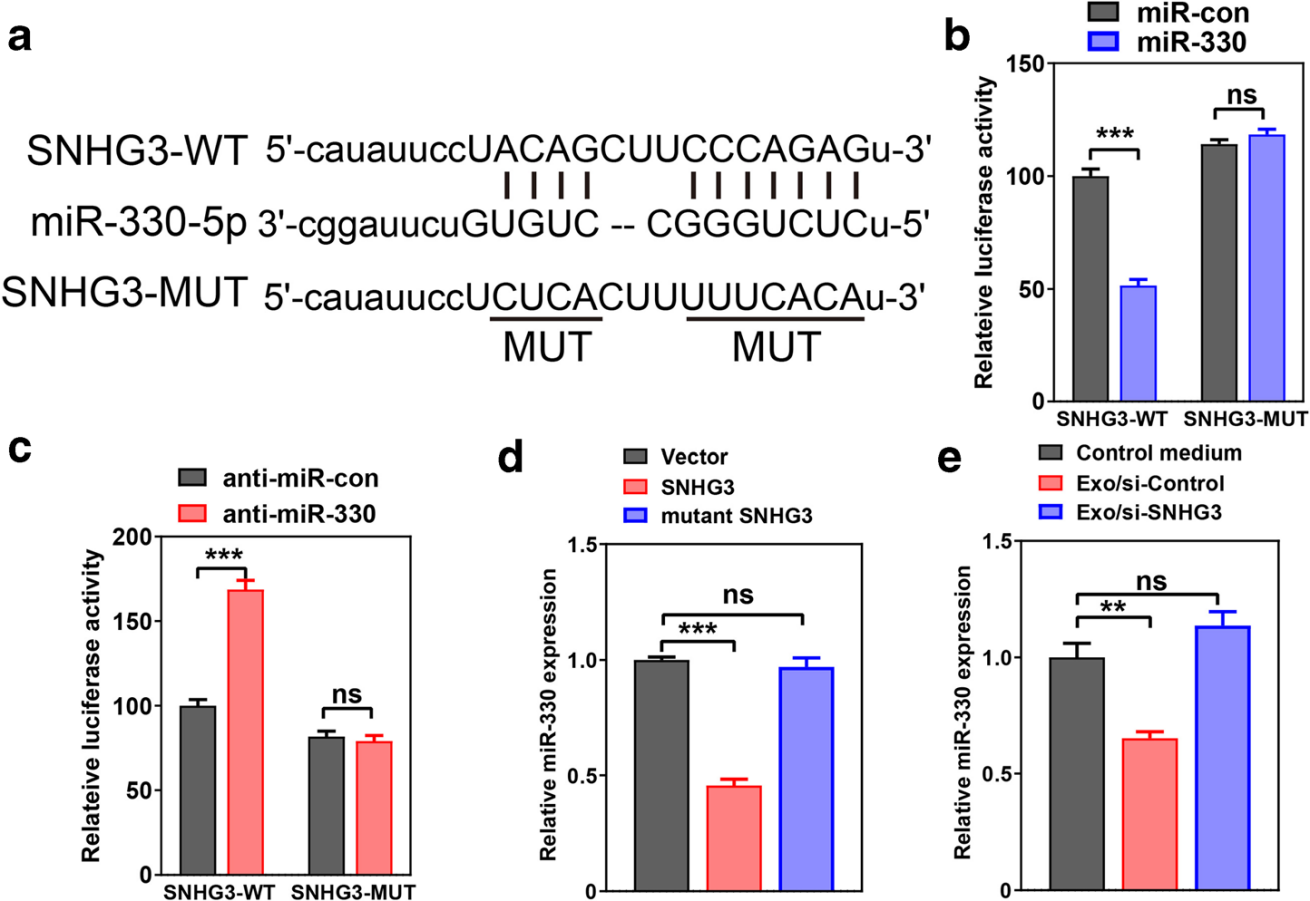
CAF-derived exosomal SNHG3 regulates the miR-330 expression in breast tumor cells. **a** The diagram illustrating luciferase reporter sequence design. **b**, **c** Luciferase activities was measured using luciferase reporter assay in MD-MBA-453 cells co-transfected with SNHG3-wildtype or SNHG3-Mutation and miR-330, anti-miR-330, or matched controls. **d** The expression of miR-330 in MD-MBA-453 cells transfected with SNHG3, mutant SNHG3 or control plasmid was measured through QPCR. **e** The expression of miR-330 was examined in MD-MBA-453 cells treated with CAF-derived exosomes transfected with si-SNHG3

Previous studies demonstrated that lncRNAs could serve as molecular sponges or ceRNAs to control the expression and function of miRNA [18–20]. To illuminate the specific mechanism by which SNHG3 exhibit oncogenic function in tumor cells, we analyzed the potential targets of SNHG3 using bioinformatics databases (miRBase and starBase). Bioinformatics analysis indicated that SNHG3 consisted of the binding sequences against the region of miR-330-5p (Fig. 3a). To further determine whether SNHG3 exerted the function of regulating miR-330 at the post-transcriptional level, dual luciferase assays were performed. The results revealed that miR-330 overexpression significantly decreased the luciferase signals of SNHG3-wildtype in MD-MBA-453 cells (Fig. 3b). However, suppression of miR-330 significantly enhanced the luciferase signals of SNHG3-wildtype in MD-MBA-453 cells, while no positive luciferase signals were observed on SNHG3-mutation (Fig. 3c). Moreover, real-time quantitative PCR showed that SNHG3 expression was significantly decreased via overexpression of SNHG3, while no significant alteration was observed in the SNHG3-mutant treatment in MD-MBA-453 cells (Fig. 3d). Exosomes secreted from CAFs with the transfection of si-SNHG3 markedly increased miR-330 expression in MD-MBA-453 cells (Fig. 3e). Taken together, our results revealed that SNHG3 suppressed the expression of miR-330 by serving as a molecular sponge in vitro.

### The Modulation of PKM Expression by SNHG3 in Breast Tumor Cells

**Fig. 4:**
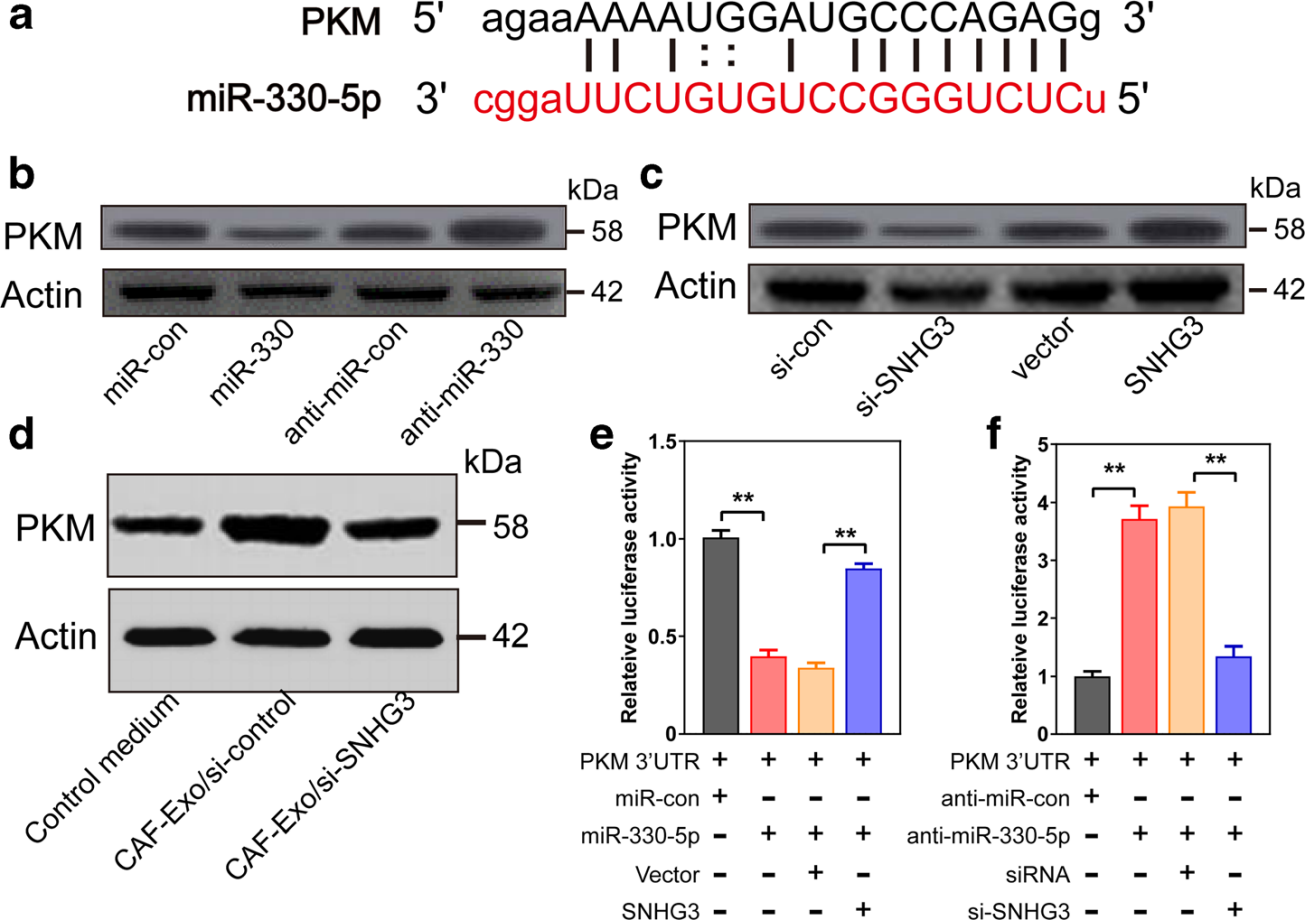
The molecular cross talk among SNHG3, PKM and miR-330. **a** Binding sites between miRNA-330-5p and PKM 3′UTR. **b** PKM protein expression in MD-MBA-453 cells transfected with miR-330, anti-miR-330, or matched controls detected using Western blot analysis. **c** PKM protein level in MD-MBA-453 cells transfected with different agents. **d** PKM protein level in MD-MBA-453cells **t**reated with CAF-derived exosomes transfected with si-SNHG3 or si-control detected by Western blot analysis. **e** The luciferase activity of MD-MBA-453 cells were analyzed by co-transfection with PKM 3’-UTR and miR-330, miR-control, miR-330 + Vector, or miR-330 + SNHG. **f** The luciferase activity of MD-MBA-453 cells were analyzed by co- transfection with PKM 3′-UTR and anti-miR-330, anti-miR-con, anti-miR-330 + si-control, or anti-miR-330 + si-SNHG3

Pyruvate kinase (PKM) functions as a key glycolytic enzyme which converts phosphoenolpyruvate to pyruvate, and the M2 isoform of pyruvate kinase (PKM2) is crucial for cancer cell metabolism, proliferation, and metastasis [21–23]. As the expression of PKM has been reported to be regulated by various miRNA at the post-transcriptional level [24, 25], we therefore investigated the effect of SNHG3/miR-330 axis on the expression of PKM and the metabolism rewiring and proliferation of breast tumor cells. First, we screened the targeting sequences between miR-330-5p and PKM mRNA using bioinformatics databases (miRBase and starBase) and showed miR-330-5p could bind to 3′UTR of PKM mRNA (Fig. 4a). Western blot showed that increasing of miR-330 and SNHG3 knockdown significantly decreased the protein expression of PKM in breast tumor cells, while silencing of miR-330 and overexpression of SNHG3 markedly increased the PKM level in MD-MBA-453 cells (Fig. 4b, c). In addition, exosomes secreted from CAFs also increased PKM expression and exosomes secreted from CAFs transfected with si-SNHG3 reversed the accumulation of PKM protein (Fig. 4d). Subsequently, miR-330 significantly suppressed the luciferase expression of PKM 3′UTR reporter activity compared to that with the miR-control treatment measured by the luciferase reporter assay, while transfection with SNHG3 markedly rescued the luciferase activity of PKM 3′-UTR reporter plasmid (Fig. 4e). On the contrary, co-transfection of MD-MBA-453 cells with anti-miR-330 and PKM 3′-UTR plasmid exhibited enhanced luciferase reporter signals, indicating the reverse by further transfection with si-SNHG3 (Fig. 4f). Collectively, our data revealed miR-330 could target PKM which was positively modulated by SNHG3.

### Knockdown of CAF-Secreted Exosomal SNHG3 Inhibited Breast Cancer Cell Proliferation Through Increasing miR-330 and Decreasing PKM Expression

**Fig. 5:**
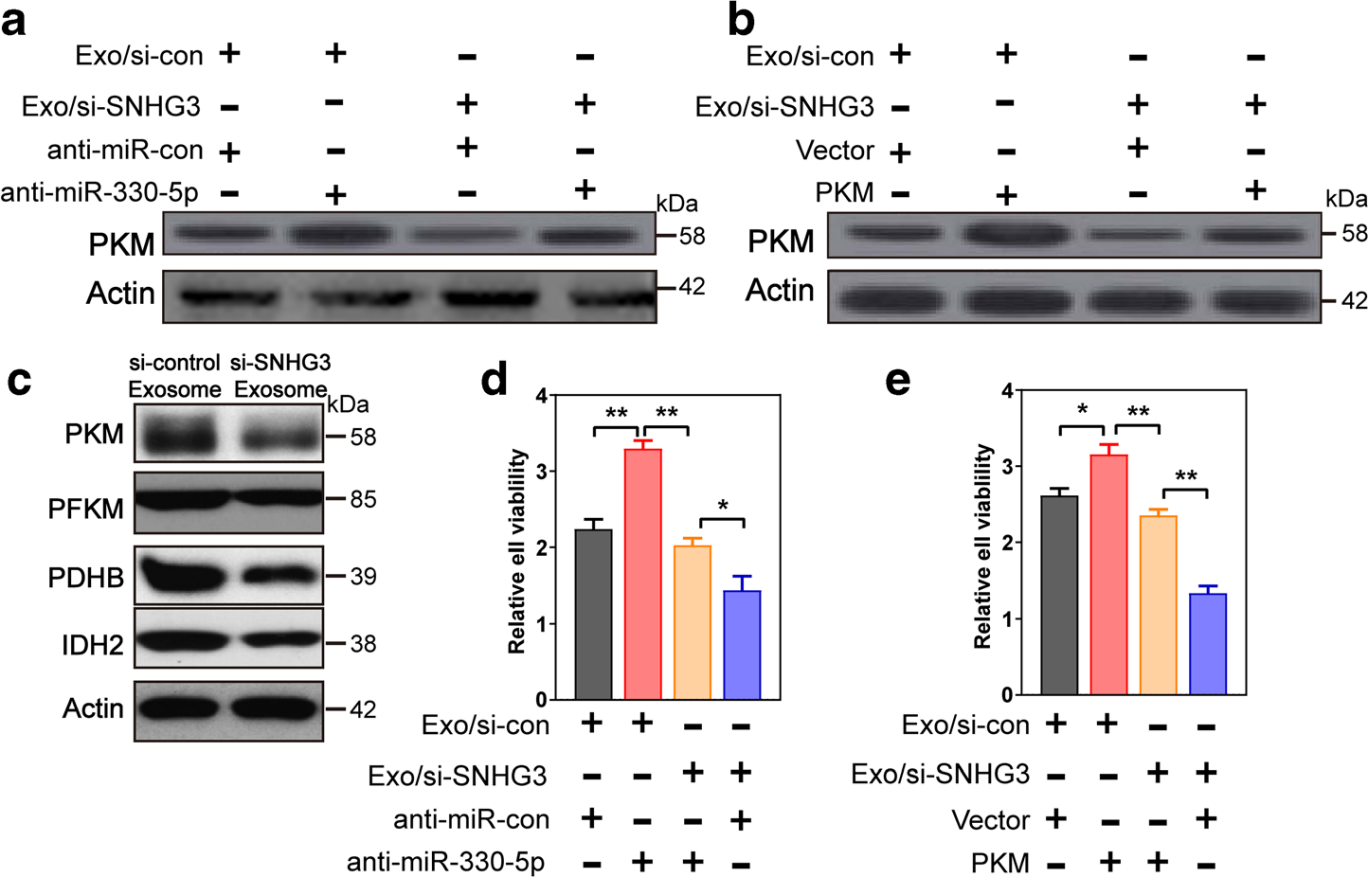
SNHG3 silencing inhibited breast cancer cell proliferation by increasing miR-330 and decreasing PKM expression. **a**, **b** MD-MBA-453 cells were transfected with si-SNHG3 or si-control, concurrently with anti-miR-330, anti-miR-control, PKM, or control plasmid. The protein expression of PKM in transfected MD-MBA-453 cells. **c** The protein expression of SNHG3-target genes and other related metabolic genes by western blot. **d**, **e** Cell proliferation was examined using CCK8 assays in MD-MBA-453 cells transfected with indicated conditions

To examine the influence of CAF-secreted exosomal SNHG3 on tumor cell proliferation through the modulation of the expression of miR-330 and PKM, MD-MBA-453 cells were co-treated with exosomes secreted from CAFs transfected by si-SNHG3 or si-control, concurrently with the transfection of anti-miR-330, anti-miR-control, PKM, or Vector. Immunoblot showed that decreasing of miR-330 (Fig. 5a) and increasing of PKM (Fig. 5b) significantly rescued CAF-secreted exosomal SNHG3 inhibition-mediated suppression of the PKM in MD-MBA-453 cells. Furthermore, the silencing of CAF-derived exosomal SNHG3 could suppress the expression of metabolism-related protein including PFKM in the glycolysis pathway and IDH2 in the Kreb’s cycle (Fig. 5c), suggesting that CAF-secreted exosomal SNHG3 may reprogram the metabolism of breast cancer cells through different molecular pathway. The CCK8 assays revealed that silencing of miR-330 (Fig. 5d) and overexpression of PKM (Fig. 5e) significantly rescued the suppression of cancer cell growth induced by the decreasing of CAF-secreted exosomal SNHG3 in MD-MBA-453 cells. Together, these results suggest that the knockdown of CAF-secreted exosomal SNHG3 inhibited breast cancer cell proliferation through increasing miR-330 and decreasing PKM expression*.*

### SNHG3 Knockdown in CAF-Derived Exosomes Inhibited Breast Cancer by the Upregulation of miR-330 and the Downregulation of PKM

**Fig. 6:**
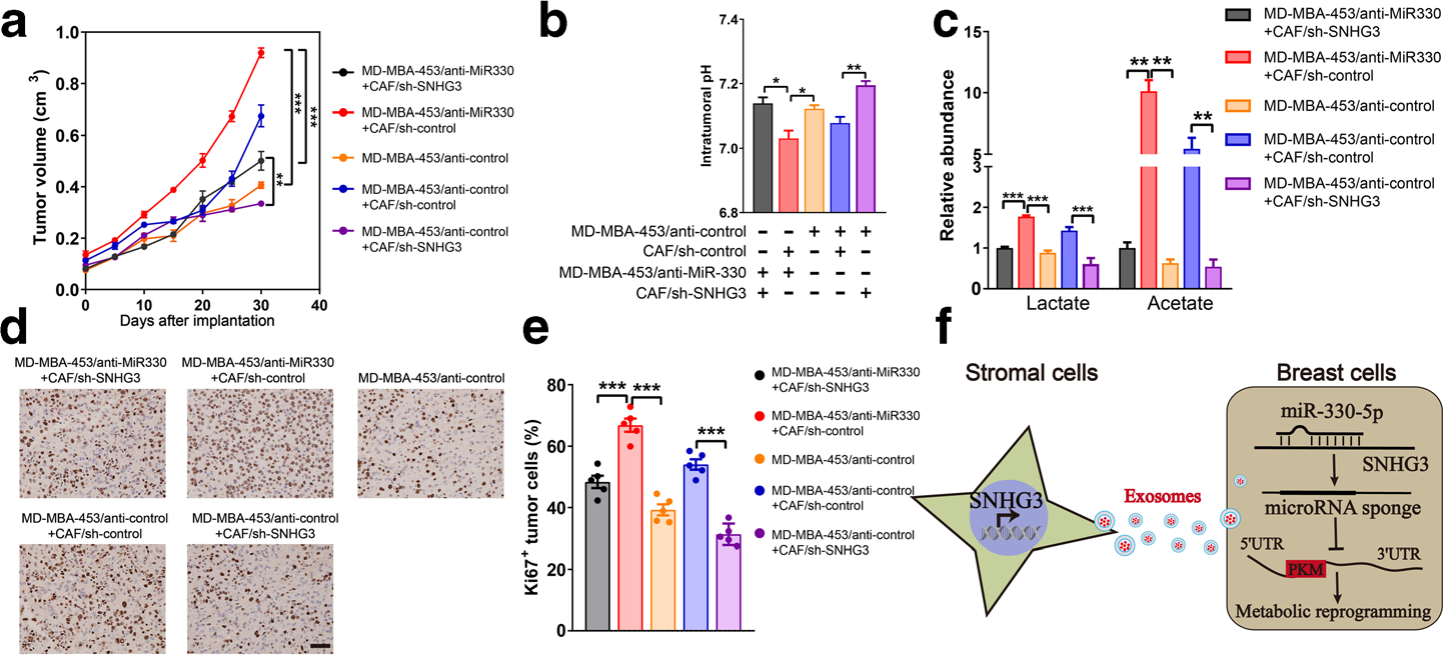
SNHG3 knockdown in CAF-derived exosomes inhibited breast tumor growth in vivo by the upregulation of miR-330 and the downregulation of PKM. **a** Breast tumor growth of mice bearing MD-MBA-453 cells (*n* = 7). **b** Tumors were isolated and detected for 1D NMR metabolic analysis (*n* = 5). **c** Intratumoral pH in the harvested tumors was measured (*n* = 5). **d**, **e** Representative Ki67 immunostaining and the positive percentage of Ki67 staining of total tumor cells (*n* = 7). **f** Working model of the metabolic reprogramming of breast cancer cells by CAF-secreted exosomes via a SNHG3-miR330-PKM-mediated mechanism identified herein

To determine whether CAF-secreted exosomal SNHG3 mediated cancer cell reprogramming results in tumor growth in vivo, we transplanted CAFs isolated from breast cancer patient which stably expressed sh-SNHG3 or sh-control, together with MD-MBA-453 cells expressing anti-miR-330 or anti-miR-control, into the breast pad of female athymic nude mice. The co-injection with breast cancer cells, expressing sh-control with the control CAFs, significantly increased tumor growth than the co-injection cancer cells expressing anti-miR-330 (Fig. 6a). Besides, the tumor growth was significantly inhibited by knockdown of SNHG3 in CAFs compared with that in the CAFs/sh-control group. Moreover, SNHG3 silencing significantly suppressed the enhanced tumor growth mediated by the inhibition of miR-330 targeting. Tumors containing CAFs/sh-control exhibited a significantly pH decline in the tumor compared with transplantation of MD-MBA-453 cells alone, and the suppression of the CAFs SNHG3 restored the tumoral pH decline (Fig. 6b). The pH decline in the group of co-transplantation of CAFs/sh-control and breast tumor cells was consistent with higher expression of metabolites including lactate and acetate (Fig. 6c) as well as increased tumor cell growth detected through Ki67 immunostaining (Fig. 6d, e). These effects were all rescued by the inhibition of SNHG3 in CAFs. In summary, our results demonstrated that knockdown of CAF-secreted exosomal SNHG3 inhibited breast cancer glycosis and growth in vivo by upregulating miR-330 and downregulating PKM.

## Discussion

Cancer-associated fibroblasts (CAFs) played an important role in tumor microenvironment in various types of solid cancers [1, 2]. Previous studies focused mainly on cell autonomous processes during tumor progression rather than investigating the communication between different types of cells in the tumor microenvironment. Accumulating researches revealed that exosomes could enhance communication between tumor and cancer-associated fibroblast cells in the tumor niche [26] and have been considered as an important cross talk pattern among different cell types in the TME [7, 16]. In this study, we provided evidences that the expression of SNHG3 was abnormally increased in breast cancer patients-derived CAFs. Knockdown of CAF-secreted exosomal SNHG3 could inhibit the growth of breast tumor cells Mechanistically, SNHG3 could serve as a molecular sponge of miR-330 to regulate the expression of PKM in breast tumor cells. These findings provide the first insights into biological function and molecular regulation of CAFs exosomal SNHG3 in breast cancer.

SNHG3 has been identified as a novel lncRNA; therefore, there were limited investigations studying the functions of SNHG3 in various tumors [10, 11, 13]. It was reported that SNHG3 was overexpressed in liver cancer, breast cancer, and colorectal cancer, which was associated with poor survival and prognosis in tumor-bearing patients [10, 11, 13, 18, 27]. Importantly, SNHG3 was identified as a potential oncogene in breast cancer based on previous studies which focused on cancer cell-autonomous function of SNHG3 [28]. Surprisingly, we found high expression of SNHG3 in exosomes secreted by CAFs isolated from breast cancer patients CAFs which may be important in the progression of breast tumor. However, the functional roles of CAF-secreted SNHG3 in breast tumor remained unknown. The results indicated that knockdown of CAF-secreted exosomal the decrease of SNHG3 expression inhibited cell growth and glycolysis metabolism of breast tumor cells in vitro and in vivo. Accumulating studies suggested that small nucleolar RNA host genes (SNHGs) could function as endogenous RNAs (ceRNAs) to regulate cancer cells by sponging miRNA, such as miR-182-5p, miR-186-5p and miR-101 [12, 27, 28]. Therefore, we proposed whether CAF-secreted exosomal SNHG3 could regulate miRNA in cancer cells [18, 28]. By bioinformatics analysis and experimental validation, SNHG3 could sponge miR-330 to promote the growth and regulate the metabolism of breast cancer cells. Notably, anti-miR-330 could partially rescue the effect of CAF-secreted exosomal SNHG3 treatment, indicating that exosomal SNHG3 may get involved in the modulation of other potential miRNAs expression. Further investigation, including the identification of novel targets of SNHG3, will be needed to define the comprehensive role of CAF-secreted exosomal SNHG3 in breast cancer.

To determine the regulation and detailed mechanism of SNHG3/miR-330 axis on the proliferation and metabolism reprograming, we performed bioinformatic analysis and predicted that miR-330 could target PKM. Pyruvate kinase muscle isozyme M2 (PKM2), one isoform of PKM, is an important glycolytic enzyme participating in the final step in glycolysis, which was essential in cancer metabolism and proliferation. Knockdown PKM could inhibit proliferation and lead to apoptosis in several different types of tumor cells [21, 22]. Besides, PKM is essential in the metabolic reprogramming, the regulation of growth, apoptosis, and metastasis of cancer cells [23]. Our study demonstrated that SNHG3/miR-330 signaling axis regulated the proliferation and metabolism of breast tumor cells through modulating PKM at the post-transcription level, providing potential therapeutic targets for inhibiting PKM in cancer treatment. Although we proved the roles of CAF-secreted exosomal SNHG3 signaling pathway in breast cancer development in vitro and in vivo, genetic mutation may still need to offer direct evidence. Therefore, knockout mouse models with conditional deletion of SNHG3 or its target miR-330 were generated and under investigation in our lab to define the exact role of SNHG3/miR-330 signaling axis in breast cancer progression.

In conclusion, our results provided novel insights into intercellular cross talk between tumor stromal and cancer cells. We demonstrated that CAF-secreted exosomes could reprogram cancer cell metabolism through the enrichment of exosomal non-coding RNA. More importantly, our results support the therapeutic potential of targeting exosomes-mediated cross talk between cancer and stromal cells in cancer treatment.
